# Pulling MscL open via N-terminal and TM1 helices: A computational study towards engineering an MscL nanovalve

**DOI:** 10.1371/journal.pone.0183822

**Published:** 2017-08-31

**Authors:** Adam D. Martinac, Navid Bavi, Omid Bavi, Boris Martinac

**Affiliations:** 1 Molecular Cardiology and Biophysics Division, Victor Chang Cardiac Research Institute, Darlinghurst, NSW, Australia; 2 St Vincent’s Clinical School, Faculty of Medicine, University of New South Wales, Darlinghurst, NSW, Australia; 3 Department of Physics, University of Tehran, Tehran, Iran; Zhejiang University Life Science Institute, CHINA

## Abstract

There are great opportunities in the manipulation of bacterial mechanosensitive (MS) ion channels for specific and targeted drug delivery purposes. Recent research has shown that these ion channels have the potential to be converted into nanovalves through clever use of magnetic nanoparticles and magnetic fields. Using a combination of molecular dynamics (MD) simulations and the finite element (FE) modelling, this study investigates the theoretical feasibility of opening the MscL channel (MS channel of large conductance of *E*. *coli*) by applying mechanical force directly to its N-terminus. This region has already been reported to function as a major mechanosensor in this channel. The stress-strain behaviour of each MscL helix was obtained using all atom MD simulations. Using the same method, we simulated two models, the wild-type (WT) MscL and the G22N mutant MscL, both embedded in a POPE lipid bilayer. In addition to indicating the main interacting residues at the hydrophobic pore, their pairwise interaction energies were monitored during the channel gating. We implemented these inputs into our FE model of MscL using curve-fitting codes and continuum mechanics equations. In the FE model, the channel could be fully opened via pulling directly on the N-terminus and bottom of TM1 by mutating dominant van der Waals interactions in the channel pore; otherwise the stress generated on the channel protein can irreversibly unravel the N-secondary structure. This is a significant finding suggesting that applying force in this manner is sufficient to open an MscL nanovalve delivering various drugs used, for example, in cancer chemotherapy. More importantly, the FE model indicates that to fully operate an MscL nanovalve by pulling directly on the N-terminus and bottom of TM1, gain-of-function (GOF) mutants (e.g., G22N MscL) would have to be employed rather than the WT MscL channel.

## Introduction

The idea to use the mechanosensitive channel of large conductance (MscL) as a nanovalve stems from the ability of MscL gating to be regulated by mechanical force transmitted through the lipid bilayer. When open, the MscL channel pore is large enough to allow the release of compounds such as anticancer drugs [1–4]. MscL is a prototype of mechanosensitive (MS) ion channels gated by bilayer tension according to the force-from-lipids paradigm [5–7]. It is able to transduce lipid deformation forces into a sequence of structural rearrangements that lead to opening of its very large pore of 2.8 nm (28 Å) in diameter [8–10]. Generating such a large pore in the membrane within a few milliseconds gives MscL a significant advantage in the design of new liposome nanovalves for controlled drug delivery.

It has previously been reported that magnetic fields could affect MscL activity [11]. In these experiments superparamagnetic particles were attached to MscL in patch clamp experiments and the channel gating was modulated by application of static magnetic field of ~300 mT. Another study showed that the N-terminus of MscL functions as a major mechanosensitive structural channel element transmitting and translating the mechanical force into the channel opening [12,13]. Moreover, it has also been shown that the MscL N-terminus resides on the lipid- water interface [12,14,15], which enables it to function as a force-conveying structural element accessible from the cytoplasmic side. These studies prompted us to use a computational approach combining MD simulations and FE modelling. While MD simulations are widely used in modelling ion channels, FE modelling has recently also been applied to a number of biological molecules, including mechanosensitive (MS) ion channels [16–24]. Combining the two approaches allowed us to estimate the conditions required to employ MscL as a liposome reconstituted nanovalve for medical or biotechnological applications.

## Materials and methods

### All-atom MD simulations

All the MD simulations were performed with NAMD2.12 [25] and Visual Molecular Dynamics (VMD) [26] was used for all visualizations and modelling G22N mutant Ec-MscL. The 3D structure of the wild type (WT) EcMscL was previously generated based on the crystal structure of MtMscL, the MscL homolog of *M*. *tuberculosis* (PDB ID: 2OAR) using Phyre [27] and Swiss-Model [12,28,29].

### Non-steered MD simulations

Both WT and G22N models were embedded into a POPE (1-palmitoyl-2-oleoyl-sn-glycero-3-phosphoethanolamine) bilayer comprised of 226 lipid molecules using VMD software. The equilibration steps have been done identically to our previous MD simulation of EcMscL [12].

Briefly, the lipids in close proximity to the channel (<5 Å) were removed first. The randomised lipid heads and tails were equilibrated for ~1 ns at 298 K, while the rest of the system was fixed. Next, the protein and lipids were solvated inside a box with dimensions 120 × 120 × 130 Å. The TIP3P water molecule model was used along with the SOLVATE program [30]. Water molecules in very close proximity to the protein (<0.5 Å) were deleted and 200 mM KCl was added to the remaining water. A further randomization of the POPE lipid tails was carried out with the rest of the system being fixed. Lipids, water and ions were packed around the protein for 1 ns and Cα atoms were restrained with a spring constant of 5 KJ/mol^−1^Å^−2^ at constant pressure of 1 atm. Finally, the whole system was equilibrated for 60 ns without restrains using a time step of 2 fs.

To open the channel surface tension of 125 mN/m was applied to the lipid molecules with a time step of 2 fs in an NγP_z_T (constant surface tension, constant pressure, and constant temperature) ensemble. The simulation times required to open the channel were ~ 16 ns (76 ns total simulation time) for the WT channel and ~ 7 ns (67 ns total simulation time) for the G22N mutant.

The particle-mesh Ewald (PME) method was used in all simulations to compute electrostatic interactions beyond a real-space cut-off of 1.2 nm using a Fourier grid spacing of 0.1 nm. A modified Nosé-Hoover Langevin piston pressure control provided in NAMD was applied to control fluctuations in the barostat around the constant pressure of 1 atm, whereas Langevin dynamics was used to control the temperature at 298 K in order to simulate the NPT ensemble. The CHARMM c36 Force field was used for all MD calculations and NAMD energy plugin was utilized for energetic calculations. The pair-wise interaction energies between two residues during the channel gating [12] were calculated using a custom tcl code.

### Steered molecular dynamics (SMD) simulation

In this study, two types of steered MD simulations (constant-velocity (CV) and constant-force (CF)) were used. The calculation of stress-strain, cross-sectional area, helix length Young’s modulus, E, determined from the spring stiffness and k in our current SMD simulations are identical to those reported previously [31].

In the constant-velocity SMD simulations, the two alpha carbons of the first two residues (e.g., N15 and V16 in the case of TM1 helix of EcMscL) on each helix were restrained using a strong harmonic restrain constant of 12 kcal/mol/Å^2^ (i.e., 840 pN/Å**)**. The last C_α_ at the opposite end of the helix called the SMD atom, is attached to another ‘dummy’ atom by a harmonic spring constant of 1 kcal/mol/Å^2^ (e.g., L48 in the TM1 helix of EcMscL) [31] All CV simulations were performed by applying the pulling rate of 0.1 Å/ps.

In the constant-force method, a constant-force vector is applied onto selected atoms on one end of the helix, while the other end is restrained. The alpha carbons of the first two residues N15 and V16 of the TM1 helix of EcMscL were restrained, whereas the force was applied on the alpha carbon of the last residue L48 in the TM1 helix of EcMscL [31]. In the case of the TM1 helix, for example, a constant-force in the range of 0.1–1.5 kcal/mol/Å (~7 to 104 pN) was applied to the C_α_ atoms of the last residue in the direction defined by a vector. The time step in all our SMD simulations was 1 fs. The pressure and temperature were controlled similar to all atom MD simulation described above. The helices were equilibrated by first holding them fixed for the first 1 ns where the atomic positions were restrained followed by their backbones being held fixed while the rest of the system was allowed to relax for the following 1 ns. Finally, the whole system was equilibrated in an NPT ensemble for 10 ns without any restraint [31]. For the SMD simulations in water, the ‘SOLVATE’ module, with the TIP3P water molecule [30] was applied in a water to a box of 40 × 40 × 90 Å.

### Finite element modelling

Finite element (FE) analysis was used to model the MscL N-terminus at the continuum level. A 3D MscL model was constructed using ABAQUS (ABAQUS FEA, Simulia, Providence, RI 02909–2499, USA) to generate data points in space based on the C_α_ coordinates obtained from the 3D crystal structure of MscL [14,32]. Note that these points do not have any properties other than position. The MscL helices were treated as elastic cylinders/rods, as previously described [12,20,33]. The Young’s moduli of each helix has been determined using steered molecular dynamics simulations [31]. The dimensions of the rods were as follows: radius r = 2.5 Å and the length of the helices, N-terminus = 18.65 Å, TM1 = 47.33 Å, TM2 = 42.51 Å, C-terminus = 36.06 Å. For meshing the TM1 and TM2 helices, C-terminus and N-terminus tetrahedral elements were used. The physiologically plausible deformation of the MscL channel was simulated by treating the rods in our model as hybrid incompressible using the Poisson’s ratio of 0.48 (almost incompressible [31,34]).

The surrounding membrane was excluded to simplify the model as the focus is purely a theoretical determination of whether the channel can be opened by pulling from the N-terminus. Additionally, by removing the lipid bilayer, the chance of singularities occurring was reduced since with the bilayer present it was difficult to apply a force or displacement to the N-terminus without the N-terminus warping the surrounding geometry and creating computational singularities, leading to distorted results.

The inner pore, consisting of the first trans-membrane helix (TM1), is constrained by a “pore-lock” comprised of a set of van der Waals forces. Although van der Waals forces are considerably weaker than the other forces which control protein conformation, the large number of van der Waals interactions can contribute significantly to protein conformation. Van der Waals interactions are comprised of two components; the complete energy of van der Waals interaction forces can be approximated by the Lennard-Jones approximation:

$$E_{vdw}\approx \frac{A_{1,2}}{{r_{1,2}}^{12}}-\frac{B_{1,2}}{{r_{1,2}}^{6}}$$ (1)

where A and B are the constants of the repulsive and attractive interactions (respectively), and r is the distance between the interacting atoms.

To obtain force contribution from the van der Waals interaction, the energy equation is derived with respect to distance:

$$F_{vdw}\approx \frac{{-12*A}_{1,2}}{{r_{1,2}}^{13}}-\frac{{6*B}_{1,2}}{{r_{1,2}}^{7}}$$ (2)

The van der Waals interactions are located between hydrophobic residues located at the narrowest part of the pore between each TM1 helix. Data obtained from the molecular dynamics simulations was used as the basis for the pore interactions (**S5 Fig**). There are 5 distinct interactions per subunit, totaling 25 interactions (5 subunits multiplied by 5 interactions) for the entire van der Waals pore lock. These interactions are between residues of neighbouring TM1 helices: (i) A20-G22, (ii) V23-G26, (iii) L19-L19, (iv) V23-V23, (v) V16-G22. The data from the molecular dynamics simulations give the interaction force between the respective residues, over a distance.

Curve fitting was applied to the MD data for each respective interactions using ‘Cftool’, the MATLAB application which provides a graphical interface where curve equations can be fit to data and plots. In the cases L19-L19, V23-G26, and V23-V23 it was found that by changing the exponents of the denominator values, better fits were obtained over the desired interaction range, Eq 2 then takes the form:

$$F_{vdw}\approx \frac{{-12*A}_{1,2}}{{r_{1,2}}^{m}}-\frac{{6*B}_{1,2}}{{r_{1,2}}^{n}}$$ (3)

Eq 3 was fit to the data sets (**S5 Fig**) and values for the variables A, B, m, and n are given in **S1 Table**.

The van der Waals interactions were implemented into the model by using connector elements, which allow for a nonlinear elastic translation of force. When set up as an elastic translating element, the connectors permit the input of a force value with a corresponding displacement value, creating a table of data points which emulate a nonlinear force in the constitutive space. The positions where the connectors were applied were based on the crystallographic sites of the respective interacting amino acid residues. The interactions appear as 5 concentric pentagonal “belts” around the inner pore of the channel **(S6 Fig)**. Displacement boundary conditions were applied to each N-terminus (±TM1) to move them from their initial closed position to a final open position.

## Results

**Fig 1:**
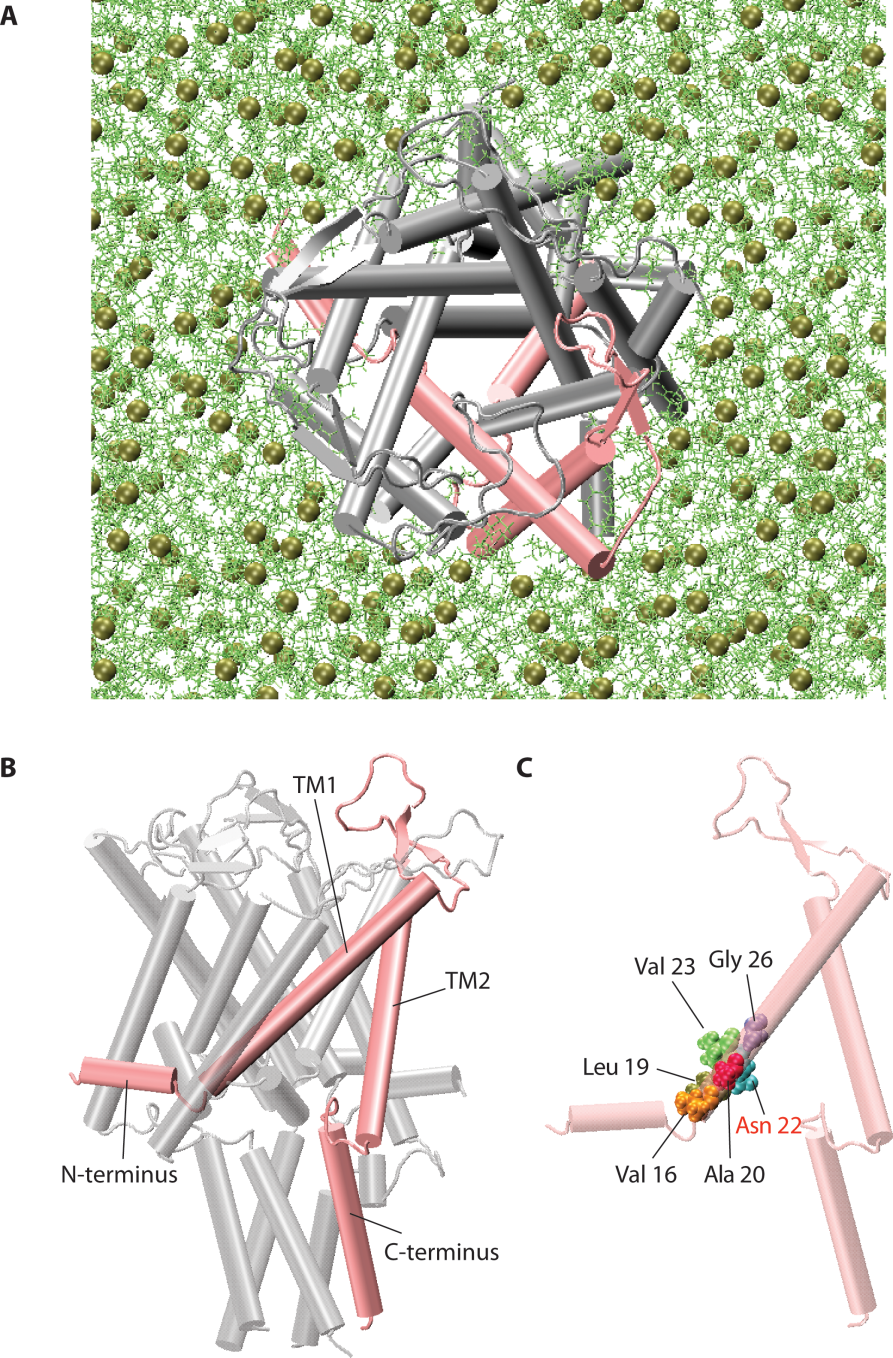
3D pentameric structure of MscL. **(A)** The three-dimensional structure of the closed (resting) state of EcMscL structure in a POPE lipid bilayer (single monomer highlighted in pink). **(B)** The MscL channel monomer is comprised of the N-terminal domain, the pore forming helix TM1, a periplasmic loop, the second transmembrane helix TM2, and the C-terminal domain facing the cytoplasm (single monomer highlighted in pink). **(C)** A single monomer showing pore lining hydrophobic residues. Note that here the Gly22 residue has been mutated to Asn22 (G22N mutant).

The aim of this study was to investigate the theoretical feasibility of opening EcMscL [35] by pulling on its N-terminus (±TM1) for its potential use as a magnetically triggered nanovalve. To determine whether WT of EcMscL is suitable, or MscL would have to be mutated for this purpose, models were created for both WT EcMscL and G22N mutant embedded in a POPE bilayer system (**Fig 1**).

**Fig 2:**
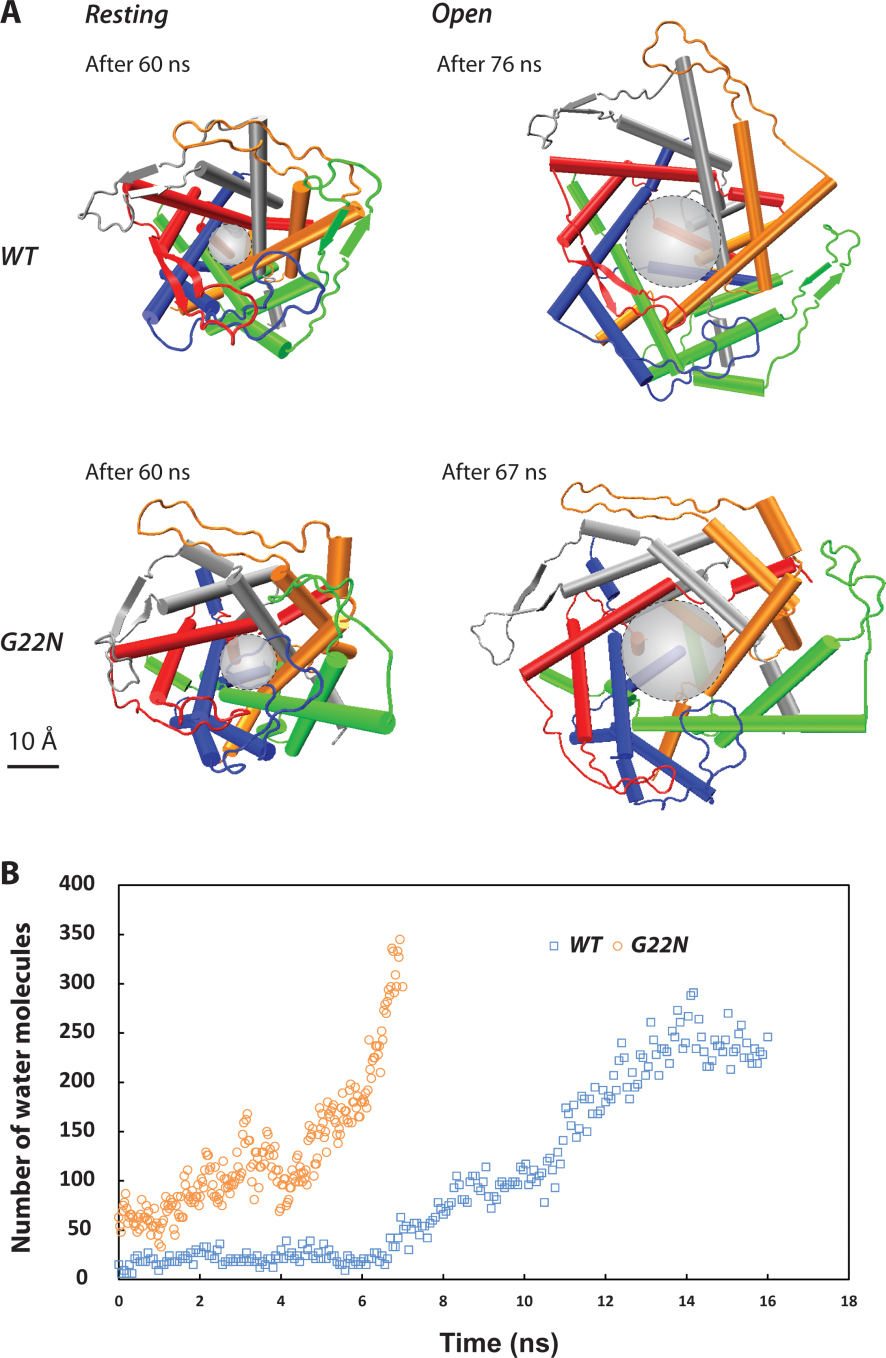
MD simulations of the WT-MscL and the G22N mutant. **(A)** By comparing the pore shape of the G22N mutant model (top view) with the WT MscL, the resting (closed) conformation of the mutant channel is wider. The (backbone to backbone) pore diameter at rest is ~ 8 Å and ~11 Å for the WT and G22N MscL, respectively. Also under similar conditions (e.g., surface tension of 125 mN/m), the G22N pore opens faster than the wild type model (~ 7 ns compared to ~ 16 ns). This result is consistent with previous experimental data showing that less force is required to activate the G22N mutant compared to WT MscL [36]. The scale bar corresponds to 10 Å. **(B)** Number of TIP3P water molecules passing by the pore lining residues (L19 to V23) during the channel gating. Unlike the WT pore, G22N pore is partially wetted (solvated) from the beginning and becomes fully wetted much faster.

In the MD simulations, as described in the methods, both MscL models (WT and G22N) were first equilibrated for 60 ns under similar conditions (**S1 Fig**) [12]. The resting conformation of both channels shows that the G22N pore is wider than the pore in the WT model (**Fig 2A**). The pore diameter of the WT model is ~ 8 Å, whilst it is ~ 11 Å in the G22N mutant (backbone to backbone distance) indicating that the G22N mutant pore is larger in the resting state. **Fig 2B** shows that there are more water molecules in the G22N pore (from residue L19 to V23) compared to the WT MscL pore. To gate the channels of both G22N and WT MD models the bilayer was stretched to overcome the gating energy barrier by increasing the surface tension of the POPE bilayer from ~ 50 mN/m, corresponding to the POPE bilayer surface tension at rest, to 125 mN/m (**Fig 2**). Under similar computational conditions, the G22N model opened faster than the WT model (7 ns versus 16 ns), indicating the predominant role of G22 residue in stabilizing the hydrophobic “pore-lock” of MscL during wetting (**Fig 2B**).

Furthermore, it was necessary to identify all pairwise inter-subunit interactions in the pore region. The interaction energy of residues 15 to 26 of each TM1 helix with their neighboring TM1 helix was screened and five pairs of residues were found to have the highest interaction energies. These residue pairs are: N22-V16, L19-L19, G22-A20 (or N22-A20 in the G22N model), V23-V23 and V23-G26, all between each two adjacent subunits (**S2 Fig**). These inter-subunit interactions form the “pore-lock” in the closed conformation of MscL. They prevent water molecules to pass through the pore.

**Fig 3:**
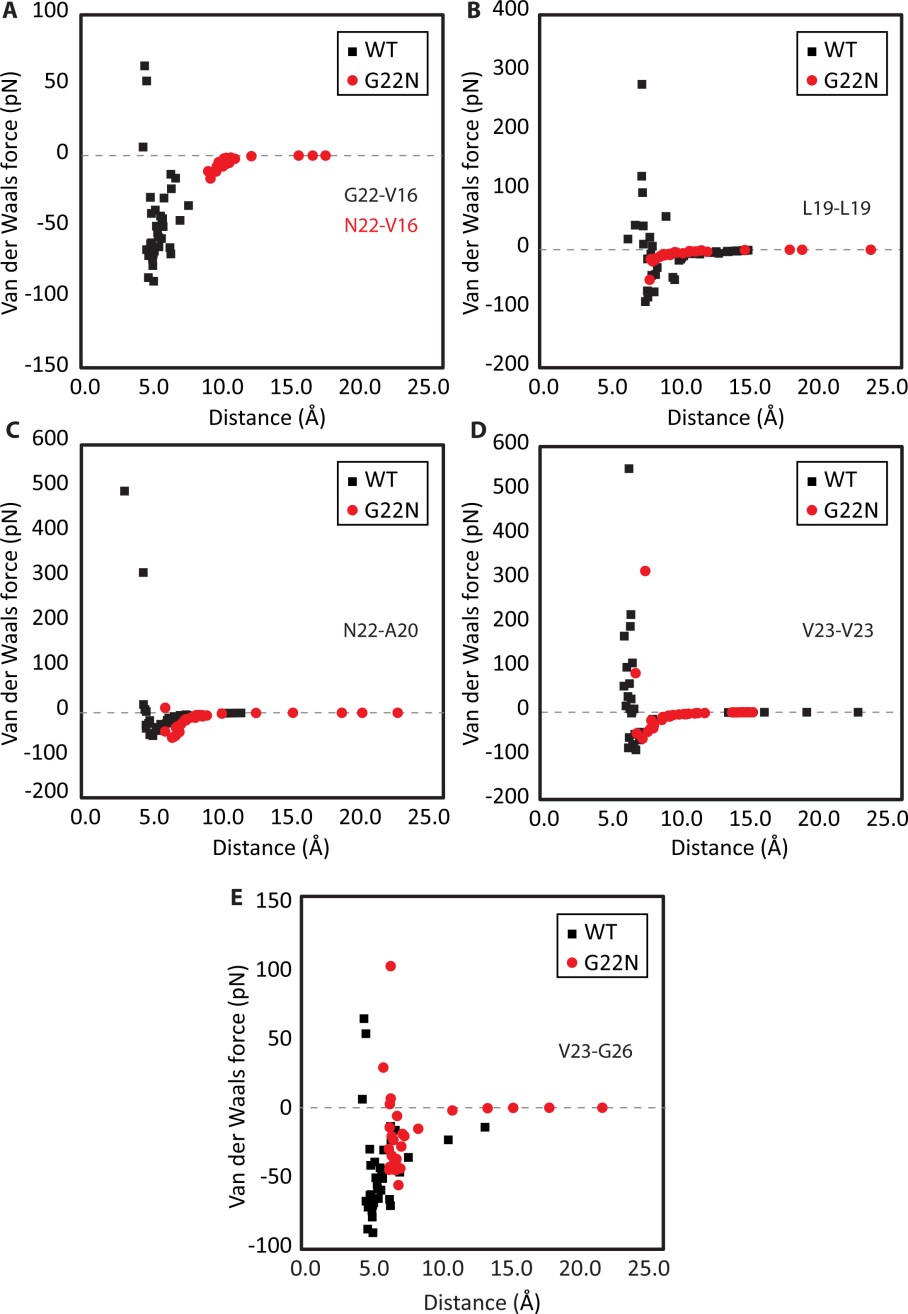
Closed-open interaction energy of the pore residues obtained from MD simulations of WT-MscL and the G22N-MscL mutant. Van der Waals (vdW) force interaction between **(A)** N22 and V16, peak to peak interaction reduction of 82% **(B)** between two L19 residues, peak to peak interaction reduction of 43% **(C)** between N22 and A20, no peak to peak interaction reduction **(D)** between two V23 residues, peak to peak interaction reduction of 31% and **(E)** between V23 and G26 of two adjacent MscL subunits, peak to peak interaction reduction of 40%. The distance shown in this figure is measured between the backbone alpha-carbon of each two residues.

In addition, the changes in the key pore interactions energies during the gating in both WT and G22N models were considered (**Fig 3**). By comparing the pore interactions of the WT MscL and the G22N mutant model, three distinct differences became apparent: i) the initial (equilibrium) distance between the pore residues in G22N are right shifted compared to those in the WT, except for the L19-L19 interaction (**Fig 3B**), ii) compared to the WT, all the pore interactions in G22N, except N22-A20, are weaker in terms of their amplitude (i.e., less negative energy values), meaning it is easier to overcome the pore interactions in G22N mutant compared to the WT MscL, and iii) from the kinetics of the interactions, three of the pore interactions in G22N MscL converge to zero considerably faster (i.e., over shorter distances) compared to those in the WT MscL (**Fig 3**). These interactions are N22-V16 (**Fig 3A**), L19-L19 (**Fig 3B**) and V23-G26 (**Fig 3E**). The kinetics of the N22-A20 and V23-V23 interactions are rather similar between the two models.

**Fig 4:**
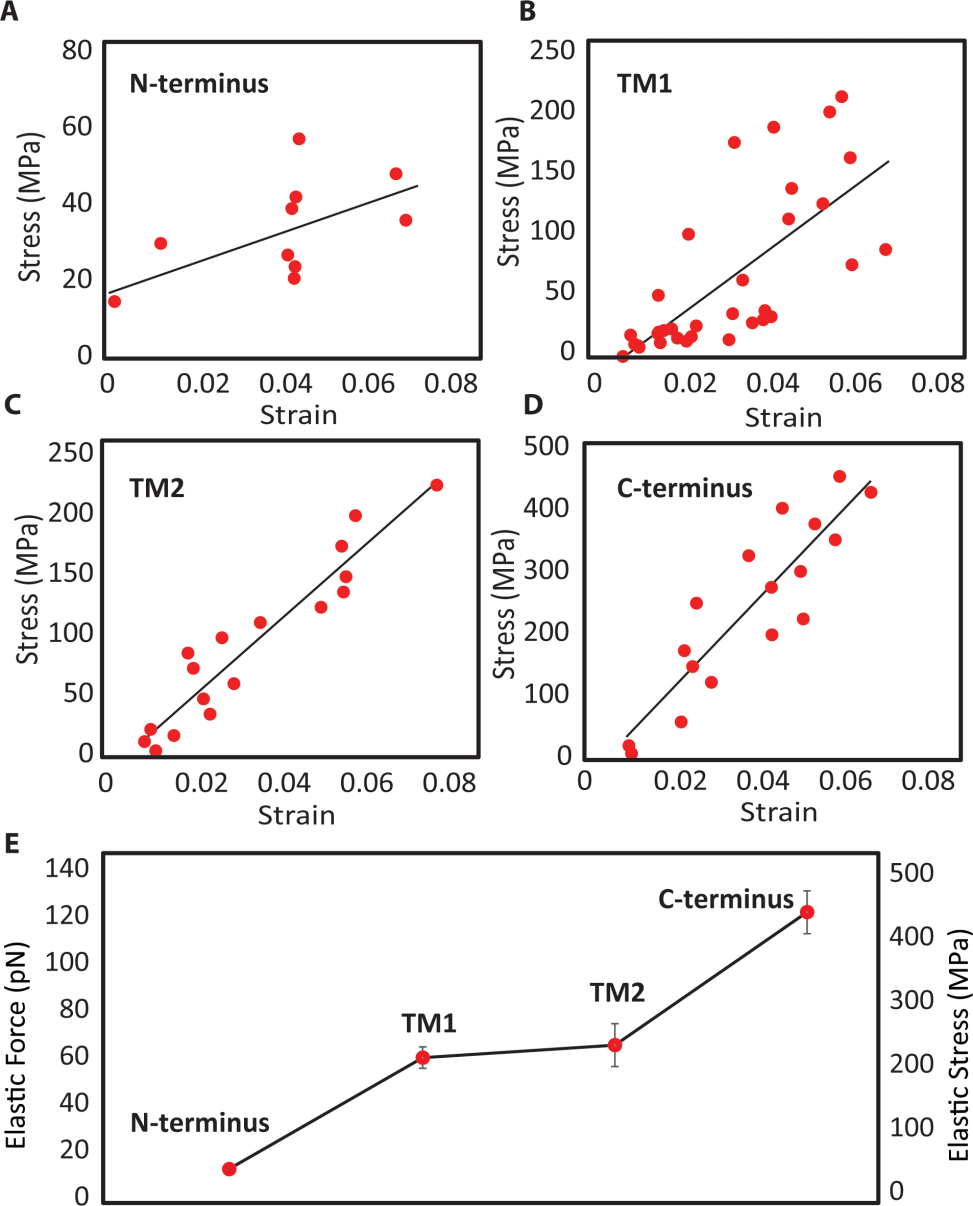
The mechanical response of EcMscL α-helices to force obtained from force-constant steered MD simulation. The stress-strain curves of **(A)** N-terminus, **(B)** TM1, **(C)** TM2, **(D)** C-terminus. **(E)** At 6% strain value, the C-terminal helix is the stiffest, whereas the N-terminal helix is the most compliant among all the α-helices. The transmembrane helices (TM1 and TM2) have similar responses, and both are more than 4 times stiffer than the N-terminus. These values are mean ± SEM of 3 different simulations.

Using constant-force steered MD simulations, we also considered the stress-strain curves of the alpha-helices EcMscL (**Fig 4**), the aim of which was to determine their potential for being the load bearing element in an MscL nanovalve model. From the response of the helices at the first 6% strain, the N-terminus (**Fig 4A**) exhibits considerably softer behavior when compared to the other helices. This indicates the N-terminus may not be capable of conveying all the directly applied force to the pore without unfolding. Therefore, the force would have to be applied to both the N-terminus and bottom of the adjacent mechanically stronger TM1 helix (**Fig 4B and 4E**) as both are accessible from the cytoplasmic side.

**Fig 5:**
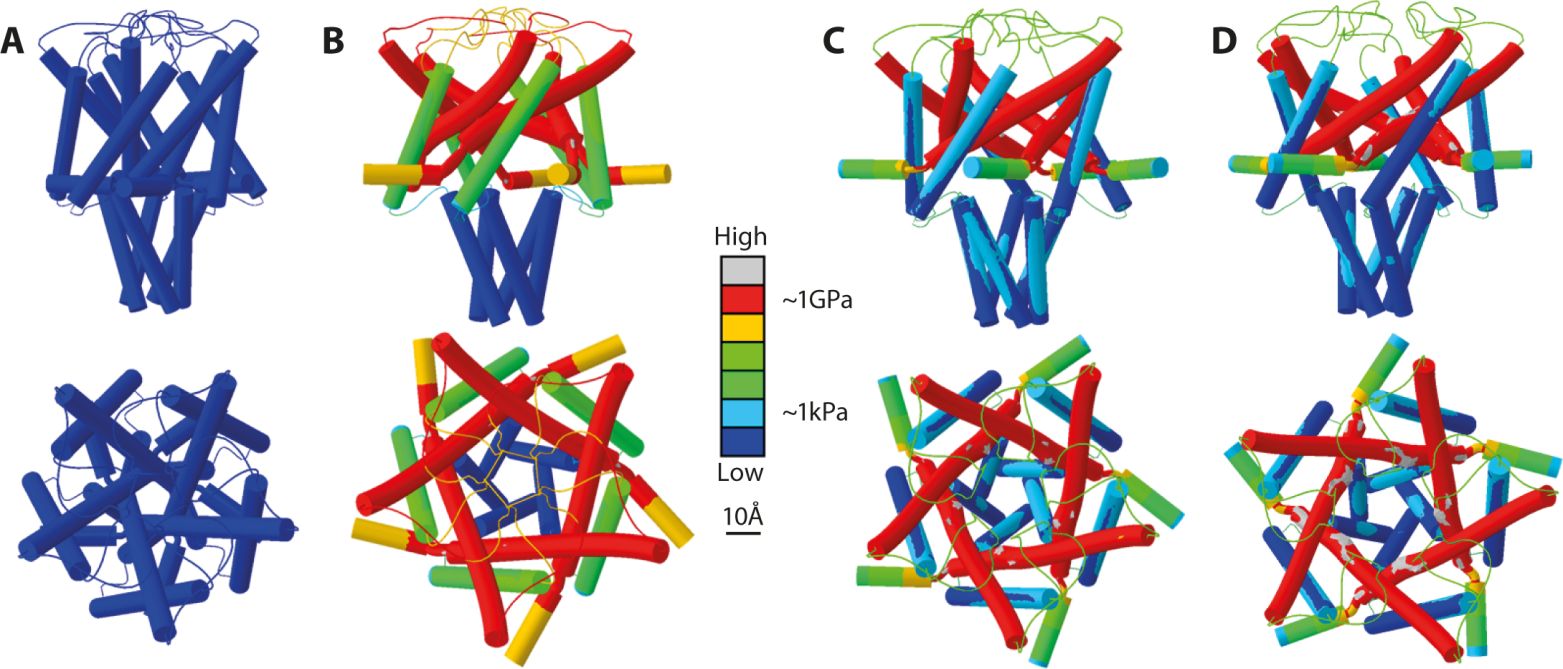
FE models of MscL. **(A)** Closed state of MscL, blue colour indicates zero force in the model. Pore diameter in closed state is 8Å. **(B)** Application of pulling force on N-terminus only with an 80% reduction of pore interactions and removal of G26-V23 interaction. Most stress is located in the TM1 helix, glycine hinge, and at the glycine hinge end of the N-terminus. The stress range along TM1 is ~30-1150MPa (1.15GPa) (**S1 Movie**). **(C)** Application of pulling force on both N-terminus and TM1 helix of WT MscL with 75% reduction of pore interaction strength. The stress range along TM1 is ~250-1800MPa (1.8GPa) (**S2 Movie**). There is significantly less stress present in the glycine hinge and the adjacent end of the N-terminus. However, due to the presence of all interactions and a lesser reduction in interaction strength, the stress along TM1 is greater than in case B. **(D)** Application of pulling force on both N-terminus and TM1 helix of G22N mutant of MscL. The stress range along TM1 is ~150-6500(6.5GPa). The scale bar corresponds to 10 Å.

Following on from the MD results FE modelling was applied to MscL. Initially, the channel pore of WT MscL was not opened by pulling on the N-terminus alone because the van der Waals pore interactions were too strong and thus counteracted the applied displacement boundary conditions (**Table 1,** in silico model 1). Consequently, the van der Waals interactions were weakened incrementally to determine whether there was a reduction in pore interaction strength where the channel would open. Simulations were developed to show a range of reductions which could open the channel by pulling directly on the N-terminus. Some of the simulation cases required the removal of entire interaction belts corresponding to a partial deletion mutant, in order to successfully simulate gating. In the case of in silico models 1 and 2 (**Table 1**), the pore diameter increased slightly from a closed state of 8Å, to 9Å and 10Å, respectively, essentially showing no change between closed and open states of the channel. Thus, in these two scenarios, there are no threshold or open state stresses. In contrast, in silico models 3–5 resulted in an open pore of 28 Å **(Table 1)**. In order to open the channel when pulling on the N-terminus a deletion of the V23-G26 interaction was necessary. As an example, **Fig 5B** shows that when pulling on the N-terminus only, the channel could be opened with an 80% reduction of the pore interactions in addition to deleting/removing the V23-G26 residue.

**Table 1:** Pore size and stress values along TM1 of several cases where pulling force was applied only to the N-terminus determined by FE modelling.

In silico model	1	2	3	4	5
**Pore Size (Å)**	9	10	28	28	25
**Threshold Stress (MPa)**	-	-	536	410	437
**Open State Stress (MPa)**	-	-	424	197	437

**Pore size** is the diameter of the channel pore, **threshold stress** is the largest average stress experienced along TM1 during the simulation (after which channel pore begins to significantly expand), the *open state stress* is the average stress along TM1 in the channel open configuration. WT configuration of simulations included all five interaction belts (25 interactions in total). The in silico models 1–5 have displacement boundary conditions applied to the N-terminus only and are distinguished as follows: **(1)** No (0%) reduction in interaction strength with a G26 deletion mutant (ΔG26), **(2)** 50% reduction and ΔG26, **(3)** 80% reduction and ΔG26, **(4)** 90% reduction and ΔG26 and, **(5)** 98% reduction with WT configuration. For images of the force distribution throughout the channel for each case see **S7 Fig**.

MscL could also be opened by pulling on the N-terminus + TM1. Similar to the N-terminus only cases, WT configuration with no reduction and a 50% reduction in interaction strength (in silico models 6 and 7, **Table 2**) resulted in an essentially unchanged pore size of 9Å. By deleting the V16-G22 and V23-G26 residue interactions from the model, in separate 50% interaction reduction simulations, the channel was opened to a pore of 26-28Å (in silico models 8 and 9, **Table 2**). In silico model 10 (**Table 2 and Fig 5C**) shows that in the WT model a 75% reduction in interaction strength led to a fully open pore. Similarly, a FE model of G22N mutant (**Fig 5D, Table 2,** in silico model 11) was also developed since G22N mutant was shown to be a GOF mutant in cell survival and patch clamp experiments [36]. The equations used for the vdW interactions (**S1 Table**), in the case of the G22N simulation, were modified by reducing their strength based on the peak to peak differences between WT and G22N MD simulations of vdW interaction energies (**Fig 3**).

**Table 2:** Pore size and stress values along TM1 of several cases where pulling force was applied to both the N-terminus and TM1.

In silico model	6	7	8	9	10	11
**Pore Size (Å)**	9	9	26	28	28	27
**Threshold Stress (MPa)**	-	-	2160	1850	807	1220
**Open State Stress (MPa)**	-	-	716	603	674	976

**Pore size** is the diameter of the channel pore, **threshold stress** is the largest average stress experienced along TM1 during the simulation (after which the channel pore begins to significantly expand), the *open state stress* is the average stress along TM1 in the channel open configuration. WT configuration of simulations included all five interaction belts (25 interactions in total). The in silico models 6–10 have displacement boundary conditions applied to both the N-terminus and the bottom TM1 and are distinguished as follows: **(6)** No (0%) reduction in interaction strength with WT configuration **(7)** 50% reduction and WT configuration, **(8)** 50% reduction and ΔV16, **(9)** 50% reduction and ΔG26 and, **(10)** 75% reduction with WT configuration. (**11)** G22N mutant with WT configuration. For images of the force distribution throughout the channel for each case see **S7 Fig**.

**Fig 6:**
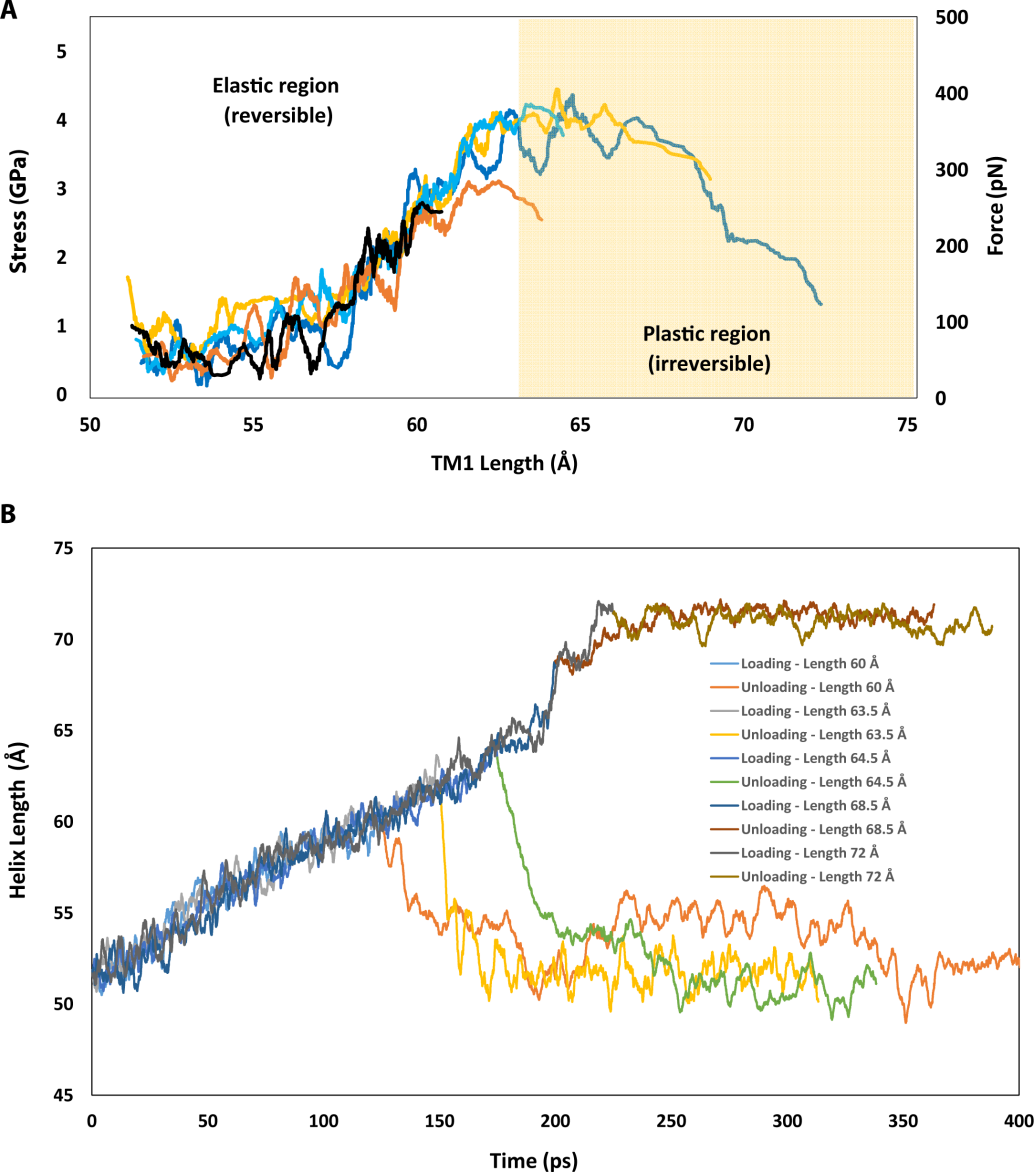
Force versus elongation of the TM1 helix. **(A)** The quasi-linear elastic (reversible) response and the plastic (irreversible) response for the TM1 helix obtained using the velocity-constant SMD simulations (see Methods). **(B)** TM1 helix exhibits a wide range of elastic deformation, up to 20% of its initial length (i.e., 20% strain). Releasing the force in the elastic region allowed the helix to go back to its initial length (elastic response), which was not the case in the plastic region; the helix undergoes a subtle elongation even after releasing the force indicating the unfolding of the helix. The elongated length of 60.0 Å in the helix corresponds to ~ 20% strain, 63.5 Å to 27% strain, 64.5 Å to 29% strain, 68.5 Å to 37% strain and, 72.0 to 44% strain.

One of the major assumptions in the FE models was that all helices have elastic properties, meaning that when the applied force is removed the helices will return to their initial shape and length (like an elastic spring). Therefore, we checked whether the stress-strains generated in the FE models during the channel gating were in an acceptable range given the load bearing capacity of each helix. Based on the FE results (75% N + TM1, in silico model 10, **Table 2**), the maximum generated stress in the TM1 helix was ~ 1 GPa (**Fig 5C and Table 2**). Consequently, it was essential to check if TM1 could handle this level of stress without permanently being unfolded. Thus, we ran a series of constant-velocity simulations which stretched the TM1 helix in a uniaxial manner using all atom MD simulations (**Fig 6**). Our results show that TM1 helix exhibits elastic behaviour (reversible) up to ~ 400 pN (~4 GPa) and up to ~ 30% of strain before unfolding irreversibly (**S3 Movie**). This simulation was repeated 5 times in the elastic region (< 40pN) to ensure that the TM1 behaviour remained elastic. Beyond the elastic force/elongation (i.e. plastic region), the helix deformed permanently, unfolding in such a manner that when the force is removed, the helix length did not return to its initial length (**Fig 5B**). These simulations were repeated at different time points, to make sure the elastic and plastic behaviour we observed were reproducible **(S3 Fig**).

## Discussion

MscL plays a crucial role in the protection of bacterial cells exposed to hypo-osmotic shock by opening a large non-selective channel pore of 28 Å in diameter that allows unhindered permeation of solvent and solutes on a millisecond time scale [37,38]. It is gated by bilayer tension and functions as a molecular transducer of mechanical stimuli acting on the bacterial cell membrane. A recent study [12] demonstrated that the horizontal amphipathic N-terminal helix of MscL functions as a crucial mechanosensory structural domain conveying membrane tension to the pore lining TM1 helix causing it to tilt and rotate in an iris-like fashion leading to the channel opening [12,15,39]. Another recent study reported modulation of activity of the MscL channel protein having superparamagnetic nanoparticles attached to it [11], which indicated the exciting possibility of converting MscL into a magnetically triggered nanovalve.

Recently, Vanegas and Arroyo, 2014 [40] applied radial pulling forces of different magnitudes on the lipids bound tightly to the N-terminus of MscL, using all-atom MD simulation. In their simulations, there are no steered forces acting directly on the protein. Here, we examined the possibility of pulling MscL open by applying force directly to its N-terminal region. To test this idea, we aimed to model the opening of MscL by pulling on the N-terminal mechanosensory amphipathic helix (±TM1 helix) and determine the conditions required to achieve this goal using a combination of FE modelling and MD simulations (FE-MD). This approach allowed us to adjust the level of detail in important regions of the channel models while remaining relatively accurate and avoiding extensive computational costs due to atomistic simulations [33,41].

### The role of the hydrophobic gate in MscL using all atom MD simulations

The importance of water dynamics within the hydrophobic pores of MS channels has been studied by a number of laboratories [42–45]. Their studies have shown that perturbing the hydrophobic pore of MscL could affect its gating characteristics, including the activation threshold and gating kinetics [42,43,46–48]. In our study, all-atom MD simulations indicated that the G22N-MscL mutant pore was much wider at rest and thus could be gated much easier than the WT in response to the membrane tension (**Fig 2A**). The G22N pore gate is less hydrophobic due to the lower energy barrier [42] and thus could accommodate a larger number of water molecules passing through the pore area during the channel gating. The MD simulations showed that wetting occurred much faster in the G22N mutant compared to the WT channel (**Fig 2B**). These observations are consistent with previous computational studies of G22N-MscL in the POPC bilayer [46,47]. Based on our results and those from previous studies, it is evident that the wetting process plays a major role in the gating of MscL.

The MD simulations identified the key pore interactions that form the “pore-lock” in MscL, which are between V16, L19, A20, G22, V23 and G26 of each two adjacent subunits (**S2 Fig**). These pore interactions are similar to those identified previously in other studies [46,48,49], except that it was found that the V23 interaction with G26 of the neighbouring subunit is quite important for constraining the MscL pore as indicated previously in a study by Iscla et al. (2013) [2]. In addition, V16-G22 was also identified as a contributing interaction (**Table 2**, in silico model 8).

Additionally, our MD simulations revealed the force range each helix can sustain before unfolding. In previous steered MD simulations, found in the literature, the range of force applied to MscL was higher (e.g. > 500 pN) than the values determined in this study (i.e. ~100pN, **Figs 5 and 6A**). To see visible changes in the protein conformation, often in MD simulations high steered forces are applied to accelerate the protein conformation process. Therefore, we employed FE modelling allowing for time-independent simulations to find more feasible steered forces.

### Opening EcMscL using finite element modelling

By combining MD data with FE modelling different scenarios of MscL opening were investigated. The van der Waals interactions within the MscL pore hydrophobic lock were too strong in the WT channel to be overcome by pulling directly on the N-terminus. Consequently, we used a progressive reduction of van der Waals interactions and determined that at 98% reduction of the initial interaction strength (**Table 1,** in silico model 5) the channel pore could be opened to ~25Å in diameter, closely corresponding to the experimentally determined diameter of the fully open MscL pore [8,38]. This level of reduction would be impractical as it would render an MscL nanovalve almost spontaneously active. Other simulations (**Tables 1 and 2**, **Fig 6**) found that there were combinations of interaction reductions and deletion mutants (80% reduction and ΔG26) which resulted in an open pore when pulling only on the N-terminus. However, in the N-terminus only cases, the stresses in the helix exceeded its elastic modulus and thus, they would cause the N-terminus to irreversibly unfold (**Figs 4 and 6**) [31]. As a result of this, it seems that in order to successfully gate the channel, without the helices unraveling, both the N-terminus and TM1 would have to be pulled together.

In addition, deletion of the G26 residue resulted in an open pore in the N-term ± TM1 helix models, despite G26 not being the locus of the strongest van der Waals interaction. Although counterintuitive at first glance, this result indicates that the constriction of the pore is also maintained higher up the TM1 helix above the G22 and V23 residues, which are located at the pore constriction (“pore-lock”) where the TM1 helices are in the closest apposition to each other. In the case where only the bottom of TM1 and/or the N-terminus is pulled, as opposed to conventional gating by membrane stretching, the force becomes concentrated in the glycine G14 hinge and towards the base of TM1. Thus, force propagation along TM1 becomes important and van der Waals interactions further up the length of TM1 exert more influence on the channel gating (**S7 Fig**). This seems, as previously shown [2], to explain the importance of the G26 residue in the channel opening mechanism, when the channel is opened by pulling on the N-terminus (±TM1 helix) rather than by membrane tension.

Furthermore, the FE modelling suggests that using MscL gain-of-function (GOF) mutants, which are easier to open because they have a lower threshold of activation by mechanical force compared to the WT channel [36], would result in significant reduction in van der Waals interaction strength. Consequently, pulling on the N-terminus ± TM1 helices of GOF mutant channels could be sufficient to fully open their pore (**Fig 5**).

### Is there an accessible force range for magnetically induced-gating of MscL?

**Fig 7:**
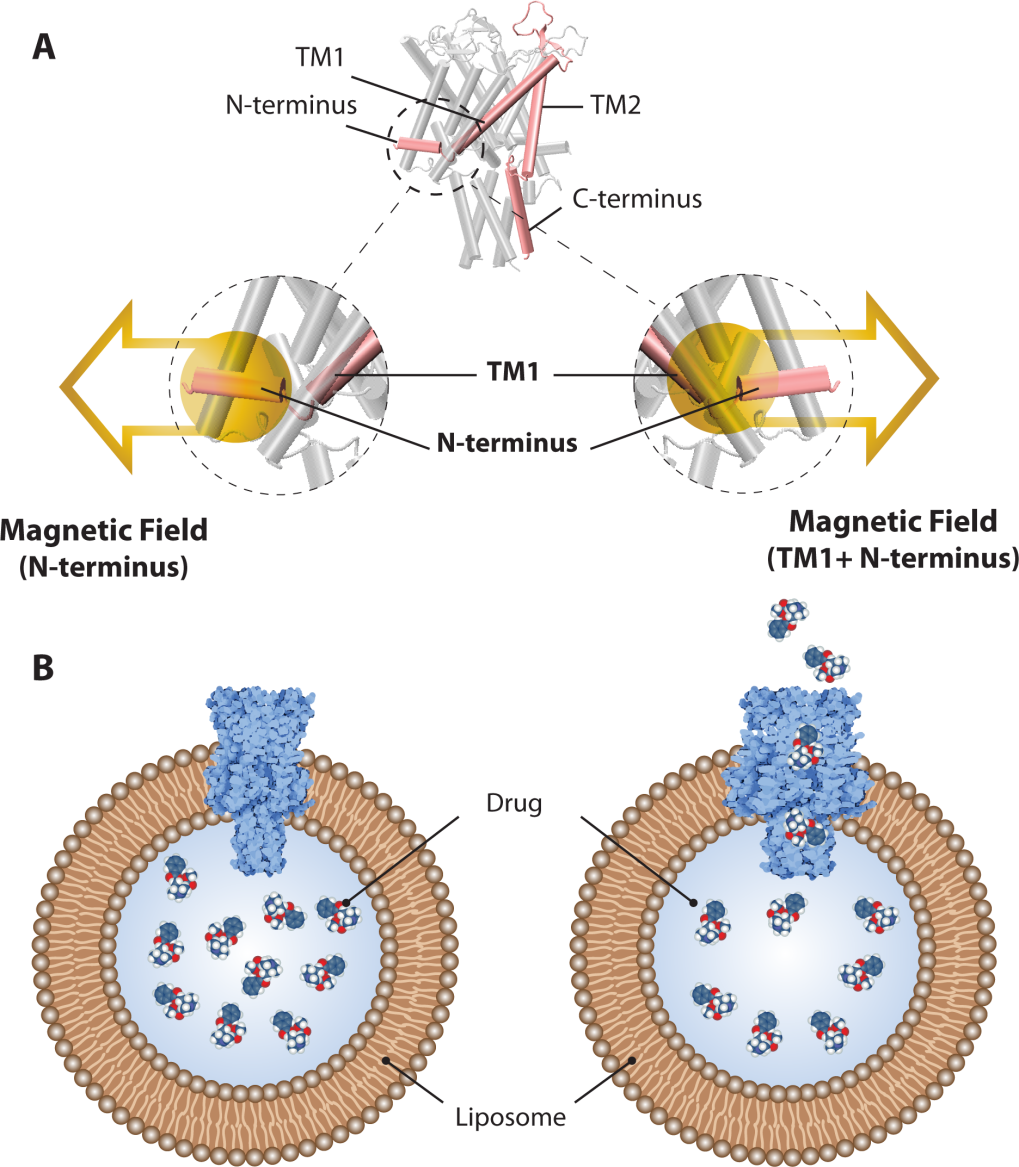
Activation of an MscL nanovalve by magnetic field. **(A)** Magnetic nano-beads are chemically attached to MscL at N-terminus (±TM1), which then can be opened by application of static and/or oscillating magnetic field to release the drug cargo carried by a liposome in which the MscL nanovalves have been reconstituted. **(B)** *Left* and *Right* images depict the closed and open MscL channel respectively, in a liposome containing drug molecules.

The idea of using MscL as a nanovalve is not new. It has been around for a number of years with one of the first being a report on a light-actuated MscL nanovalve [50]. Based on some of our recent experimental results [11], we have entertained the idea of a magnetically triggered MscL nanovalve in liposomal systems where using magnetic fields (**Fig 7**) as a trigger would be more advantageous. In particular, it could be beneficial in cases such as the treatment of different types of cancers, given that biological materials, including the human body, are more transparent to magnetic fields compared to visible light. This would open up a possibility of releasing liposome cargo at specific disease target sites inside the body of a patient.

Within this context an important question to ask is how strong a magnetic field should be in order to activate a magnetically triggered MscL nanovalve. To turn MscL into such a nanovalve, magnetic nanoparticles have to be chemically attached to the N-terminus ± TM1 of MscL (**Fig 7**) so that the channel can be opened by application of a magnetic field. Superparamagnetic iron oxide nanoparticles (SPIONs), synthetic maghemite or magnetite particles between 10 and 100 nm in size, have been used, for over 10 years, as drug delivery vehicles [51]. Given their superparamagnetic properties they can be magnetized by an external magnetic field up to their saturation magnetization, which upon removal of the magnetic field fades away. These particles are usually coated with dextran or polyethylene glycol, which allow the conjugation of therapeutic agents with their surface. Similarly, cobalt-iron superparamagnetic nanoparticles can be coated with hexanedithiol to attach the particles via sulfhydryl chemistry to MscL; one residue is mutated to cysteine for this purpose [11]. It has been reported that a magnetic flux density of ~200–400 mT, characteristic of strong permanent neodymium (NdFeB) magnets at a distance of 30 mm from the magnet pole, is sufficient to direct the movement of the superparamagnetic particles [52]. This finding is in accordance with the results of a study showing that activity of MscL channels, with nanoparticles attached increased upon application of a magnetic field of 300 mT [12]. Together, these results indicate a good possibility of generating and employing MscL as a nanovalve in liposomal preparations for drug delivery.

## Conclusions

We investigated van der Waals interactions between pore residues in the MscL channel of *E*. *coli*. The MD simulations and FE modelling in this study indicate that these interactions are too strong to open the WT MscL channel by pulling on its N-terminus alone. MscL can, nevertheless, be fully opened by pulling on the N-terminus in conjunction with the bottom of TM1 if the van der Waals interactions between the pore residues are weakened by mutating these residues, examples being a 75% reduction and the GOF G22N-MscL channel mutant **(Fig 5C and 5D)**. This finding suggests the feasibility of employing GOF MscL mutants as MscL nanovalves in liposomal preparations delivering various drugs for clinical use such as in cancer chemotherapy.

## Supporting information

S1 Fig Root Mean Square Deviation (RMSD) of the protein backbone carbons plotted against simulation time for wild type (WT) Ec-MscL and G22N mutant in a POPE lipid bilayer. Both WT and mutant models are well equilibrated after 60 ns.
(TIF)

S2 Fig Pairwise inter-subunit interactions of the pore lining residues in G22N mutant MscL. Note the pore lining residues are the same for the WT MscL but with slightly different residue to residue distances.
(TIF)

S3 Fig Loading and unloading of the TM1 helix of EcMscL. The unloading step was repeated at 29% helical strain (i.e., helix length elongated to 64.5 Å; black trace) using constant-velocity SMD simulation (see methods). When the force is released, the helix recovers its initial length (elastic response). The unloading simulation was repeated twice. The repeats are shown with grey and yellow traces and all resulted in similar responses. The 1st run is shown with grey, and the 2nd run with yellow.
(TIF)

S4 Fig Loading and unloading of the TM1 helix of EcMscL. The unloading step was repeated after ~ 37% helical strain (i.e., helix elongates to 68.5 Å; black trace). We used constant-velocity SMD simulation (see methods). When the force was released, the helix further elongated for another ~ 4 Å and then its length remained unchanged (plastic response). The unloading simulation was repeated three times and all resulted in similar responses. The 1st run is shown with grey, the 2nd run with yellow and the third one with a blue.
(TIF)

S5 Fig Curve fit of L19-L19 van der Waals interactions in MscL. Molecular dynamics simulation data of van der Waals interactions between L19 and L19 (orange). Curve fit of MD data (blue line). Curve fitting was applied to the MD data for each respective interactions using Cftool, the MATLAB application which provides a graphical interface where curve equations can be fit to data and plots. Eq 3 in the main text was fit to each set of data.
(TIF)

S6 Fig FE model of the MscL “pore-lock”. Top (top panel) and angled views (bottom panel) of “pore-lock” structure with van der Waals interactions implemented into the FE model via connector elements.
(TIF)

S7 Fig All FE simulations conducted in this study. **(A)** Closed MscL channel state. **(B)** No reduction in van der Waals (vdW) interaction strength and ΔG26, pulling only on the N-terminus. **(C)** 50% reduction in vdW interaction strength and ΔG26, pulling only on the N-terminus. **(D)** 80% reduction in vdW interaction strength and ΔG26, pulling only on the N-terminus. **(E)** 90% reduction in vdW interaction strength and ΔG26, pulling only on the N-terminus. **(F)** 98% reduction in vdW interaction strength and ΔG26, pulling only on the N-terminus. **(G)** No reduction in vdW interaction strength, pulling on both the N-terminus and bottom of TM1. **(H)** 50% reduction in vdW interaction strength, pulling on both the N-terminus and bottom of TM1. **(I)** 50% reduction in vdW interaction strength and ΔV16, pulling on both the N-terminus and bottom of TM1. **(J)** 50% reduction in vdW interaction strength and ΔG26, pulling on both the N-terminus and bottom of TM1. **(K)** 75% reduction in vdW interaction strength, pulling on both the N-terminus and bottom of TM1. **(L)** G22N mutation, pulling on both N-terminus and bottom of TM1. For pore size and stress values along TM1 see Tables 1 and 2 in the main text. Also note the different stress distributions in each case along the N-terminal and TM1 helices.
(TIF)

S1 TableValues for variables A, B, m, and n of each van der Waals interaction determined by curve fitting.Variables for the following equation used to fit MD data: $F_{vdw}\approx \frac{-12\times A_{1,2}}{{r_{1,2}}^{m}}+\frac{{6\times B}_{1,2}}{{r_{1,2}}^{n}}$.(DOCX)

S1 Movie Opening MscL by pulling on N-terminus alone. Pulling force was applied to the N-terminus only with 80% reduction in pore interaction and removal of G26-V23 interaction. Note that the highest stress is located in the glycine-TM1 hinge. The stress range along TM1 is ~30-1150MPa (1.15GPa).
(AVI)

S2 Movie Opening MscL by pulling on N-terminus together with the bottom of TM1. Pulling force was applied to both N-terminus and TM1 helix of WT MscL with 75% reduction in pore interaction strength. The stress range along TM1 is ~250-1800MPa (1.8GPa). Note that the highest stresses are located at the connector end points between the helices.
(AVI)

S3 Movie Elongation of the TM1 helix using constant-velocity steered MD simulation. In this simulation the end-most segment of the TM1 helix (the pore lining helix in EcMscL) has been stretched to obtain its mechanical response to the axial force. **(A)**The movie shows the quasi-linear elastic (reversible) response and **(B)** the plastic (irreversible) response of the TM1 helix at 10% strain and 44% strain, respectively. The elastic force-elongation response of the TM1 helix is shown in Fig 5. The hydrogen bonds on the TM1 helix of EcMscL during 200 ps simulation are shown with cyan beams. The details of this simulation have been described in the methods.
(MP4)
