# Supplementary figures and images for: Pulling MscL open via N-terminal and TM1 helices: A computational study towards engineering an MscL nanovalve
Source: PLoS One. 2017 Aug 31;12(8):e0183822. doi: 10.1371/journal.pone.0183822 (PMC5578686; doi:10.1371/journal.pone.0183822)

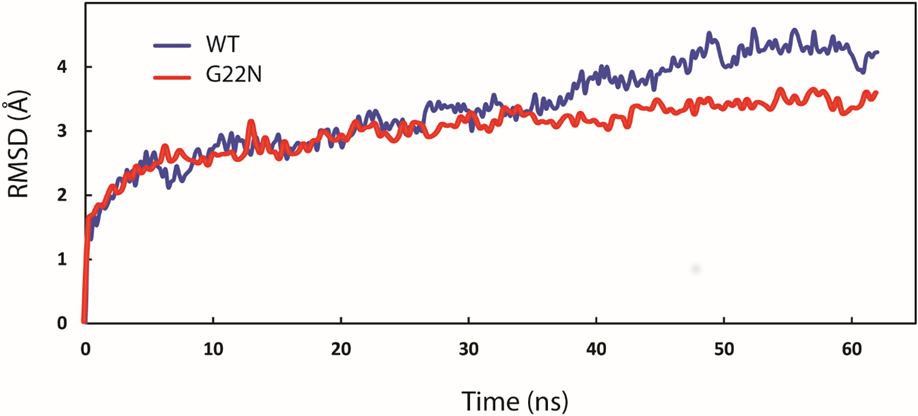

Supplement: S1 Fig — Both WT and mutant models are well equilibrated after 60 ns. (TIF) [file pone.0183822.s001.tif]

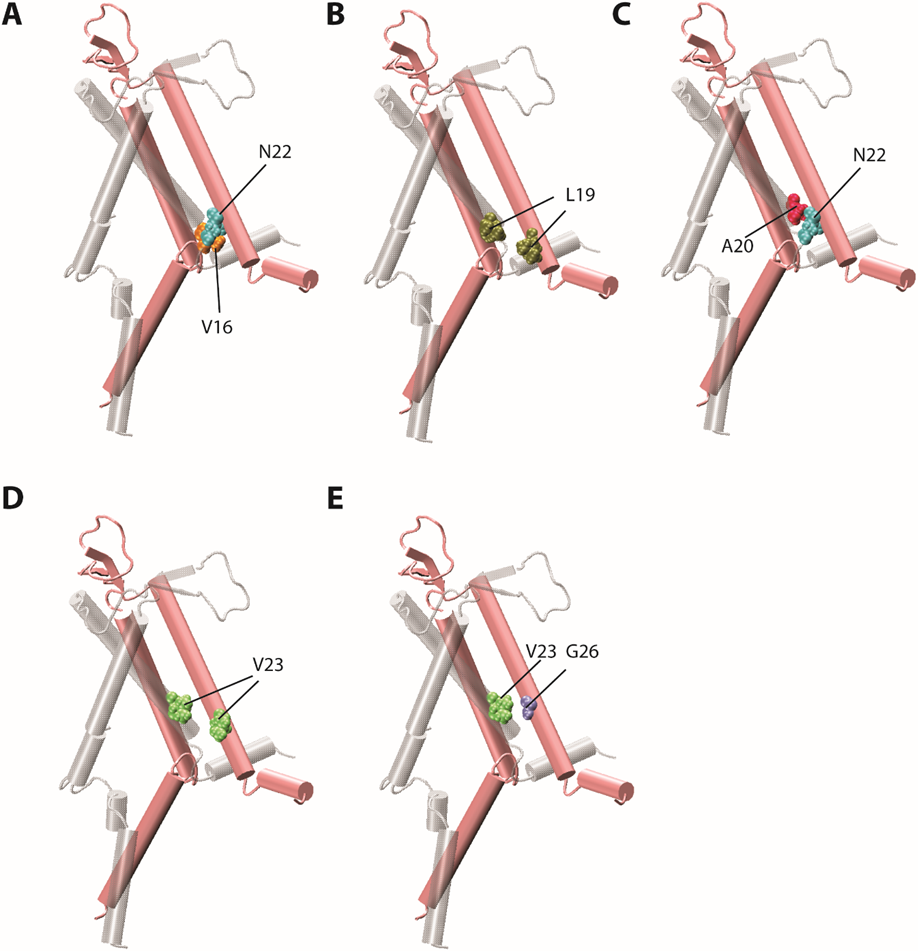

Supplement: S2 Fig — Note the pore lining residues are the same for the WT MscL but with slightly different residue to residue distances. (TIF) [file pone.0183822.s002.tif]

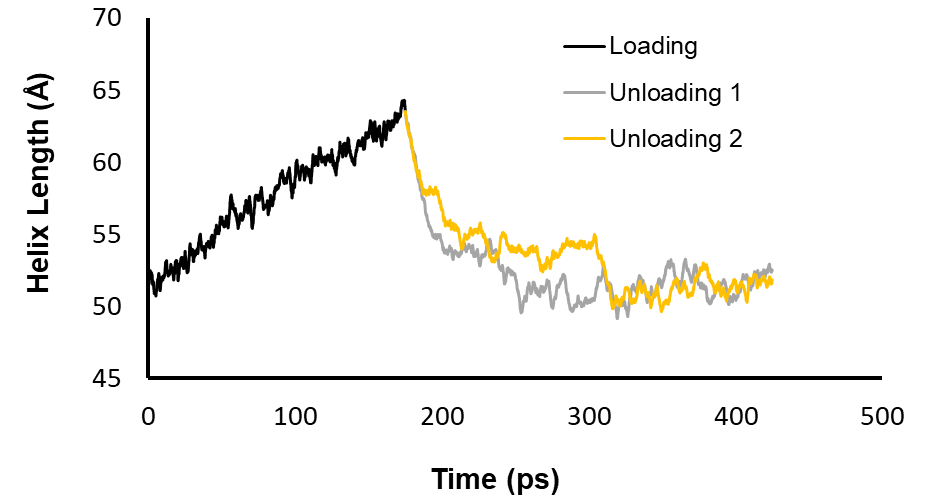

Supplement: S3 Fig — The unloading step was repeated at 29% helical strain (i.e., helix length elongated to 64.5 Å; black trace) using constant-velocity SMD simulation (see methods). When the force is released, the helix recovers its initial length (elastic response). The unloading simulation was repeated twice. The repeats are shown with grey and yellow traces and all resulted in similar responses. The 1st run is shown with grey, and the 2nd run with yellow. (TIF) [file pone.0183822.s003.tif]

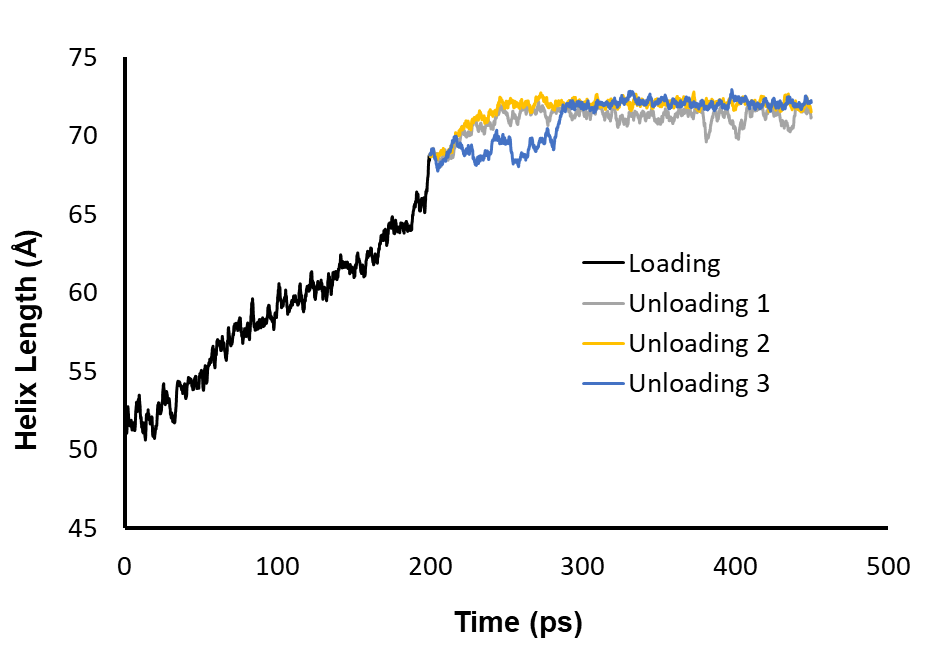

Supplement: S4 Fig — The unloading step was repeated after ~ 37% helical strain (i.e., helix elongates to 68.5 Å; black trace). We used constant-velocity SMD simulation (see methods). When the force was released, the helix further elongated for another ~ 4 Å and then its length remained unchanged (plastic response). The unloading simulation was repeated three times and all resulted in similar responses. The 1st run is shown with grey, the 2nd run with yellow and the third one with a blue. (TIF) [file pone.0183822.s004.tif]

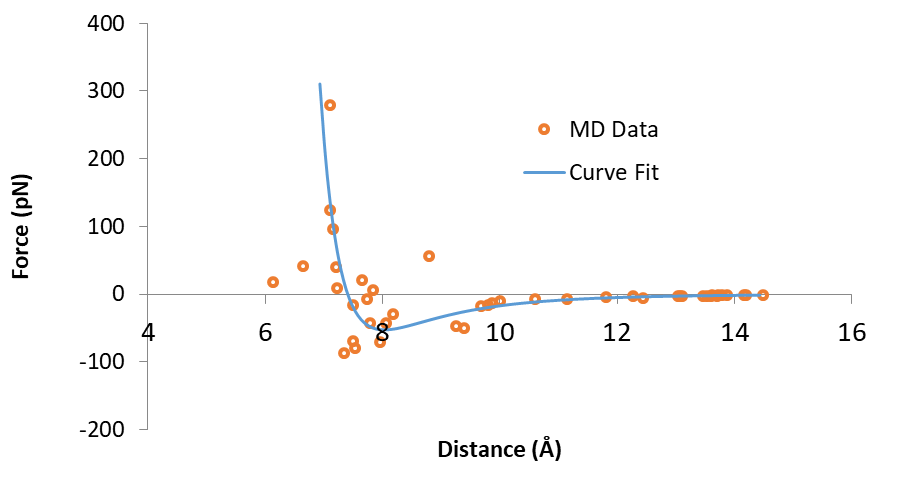

Supplement: S5 Fig — Molecular dynamics simulation data of van der Waals interactions between L19 and L19 (orange). Curve fit of MD data (blue line). Curve fitting was applied to the MD data for each respective interactions using Cftool, the MATLAB application which provides a graphical interface where curve equations can be fit to data and plots. Eq 3 in the main text was fit to each set of data. (TIF) [file pone.0183822.s005.tif]

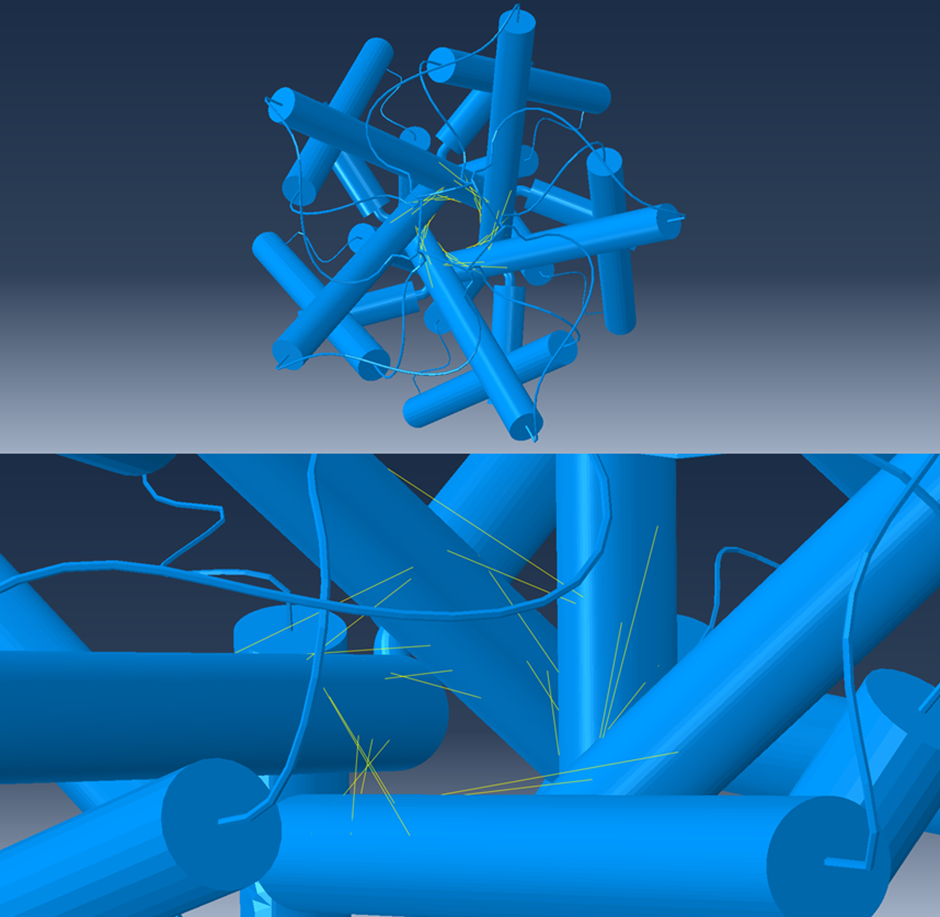

Supplement: S6 Fig — Top (top panel) and angled views (bottom panel) of “pore-lock” structure with van der Waals interactions implemented into the FE model via connector elements. (TIF) [file pone.0183822.s006.tif]

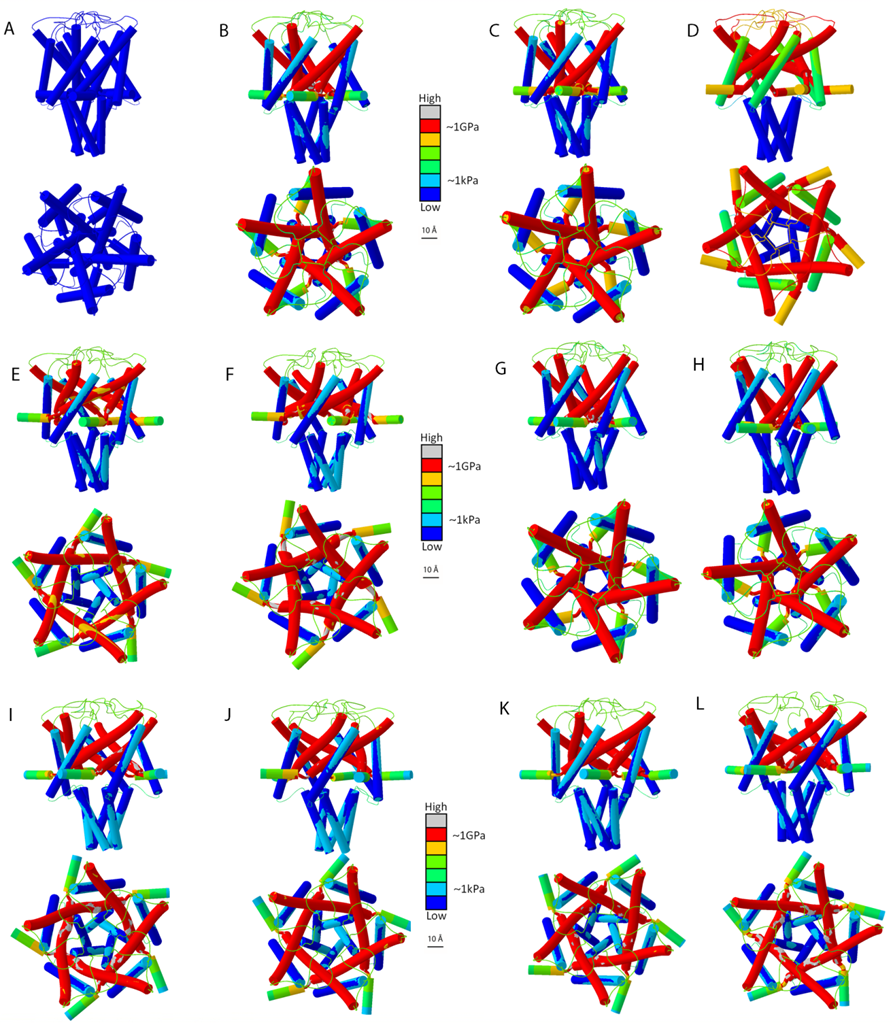

Supplement: S7 Fig — (A) Closed MscL channel state. (B) No reduction in van der Waals (vdW) interaction strength and ΔG26, pulling only on the N-terminus. (C) 50% reduction in vdW interaction strength and ΔG26, pulling only on the N-terminus. (D) 80% reduction in vdW interaction strength and ΔG26, pulling only on the N-terminus. (E) 90% reduction in vdW interaction strength and ΔG26, pulling only on the N-terminus. (F) 98% reduction in vdW interaction strength and ΔG26, pulling only on the N-terminus. (G) No reduction in vdW interaction strength, pulling on both the N-terminus and bottom of TM1. (H) 50% reduction in vdW interaction strength, pulling on both the N-terminus and bottom of TM1. (I) 50% reduction in vdW interaction strength and ΔV16, pulling on both the N-terminus and bottom of TM1. (J) 50% reduction in vdW interaction strength and ΔG26, pulling on both the N-terminus and bottom of TM1. (K) 75% reduction in vdW interaction strength, pulling on both the N-terminus and bottom of TM1. (L) G22N mutation, pulling on both N-terminus and bottom of TM1. For pore size and stress values along TM1 see Tables 1 and 2 in the main text. Also note the different stress distributions in each case along the N-terminal and TM1 helices. (TIF) [file pone.0183822.s007.tif]
