# Supplementary material for: Pulling MscL open via N-terminal and TM1 helices: A computational study towards engineering an MscL nanovalve
Source: PLoS One. 2017 Aug 31;12(8):e0183822. doi: 10.1371/journal.pone.0183822 (PMC5578686; doi:10.1371/journal.pone.0183822)
Supplement: S1 Table — Variables for the following equation used to fit MD data: Fvdw≈−12×A1,2r1,2m+6×B1,2r1,2n. (DOCX) [file pone.0183822.s008.docx]

**S1 Table: Values for variables A, B, m, and n of each van der Waals interaction determined by curve fitting.**

|  | A20-G22 | V23-G26 | L19-L19 | V23-V23 | V16-G22 |
| --- | --- | --- | --- | --- | --- |
| $\mathbf{A}_{\mathbf{1,2}}$ | -0.004073 | -6.203e4 | -2.921e6 | -1.502e41 | -0.005412 |
| $\mathbf{B}_{\mathbf{1,2}}$ | -1.037e-6 | -2.682e-4 | -2.9e-5 | -0.4191 | -1.518e-6 |
| m | 13 | 20 | 20 | 60 | 13 |
| n | 7 | 9 | 7 | 12 | 7 |

Variables for the following equation used to fit MD data: $F_{\mathrm{vdw}}\approx\frac{-12\times A_{1,2}}{{r_{1,2}}^{m}}+\frac{{6\times B}_{1,2}}{{r_{1,2}}^{n}}$
